# Frequency of KIT Mutation in Gastrointestinal Stromal Tumors According to Histologic and Immunohistochemical Findings, the First Report from Iran

**Published:** 2015-07

**Authors:** Bita Geramizadeh, Zahra Jowkar, Zeinab Ranjbar

**Affiliations:** 1 Transplant Research Center, Department of Pathology, School of Medicine, Shiraz University of Medical Sciences, Shiraz, Iran;; 2 Department of Pathology, School of Medicine,Shiraz University of Medical Sciences, Shiraz, Iran

**Keywords:** Immunohistochemistry, Gastrointestinal stromal tumor, KIT mutation, Iran

## Abstract

**Background:**

Gastrointestinal stromal tumor (GIST) is the most common mesenchymal neoplasm of the gastrointestinal tract. They are believed to originate from the interstitial cells of Cajal. Most of these tumors contain activating mutations in the KIT receptor tyrosine kinase. This is the first study in Iran to evaluate GISTs at the molecular level.

**Methods:**

In the present study, during 5 years (2007-2012), we found 50 cases of GISTs (recurrent or treated cases have been omitted) from the affiliated hospitals of Shiraz University of Medical Sciences. Demographic findings and gross characteristics have been extracted from the clinical charts and pathology reports, respectively. In addition, immunohistochemistry for c-KIT and DOG-1 were performed and reviewed by two pathologists. Molecular study for two common exons of KIT (9,11)  were performed by PCR and bidirectional DNA sequencing.

**Results:**

Among 50 cases of GIST, 17 cases showed wild type KIT and 33 cases (66%) with mutation either in exon 9 or in exon 11. The mutation of exon 9 was detected in 11 (22%) cases, while 29 (58%) cases had mutation of exon 11. In seven cases, both exon 11 and exon 9 mutations were detected at the same time (14%).

**Conclusion:**

There is significant variation in the frequency of KIT mutation in exon 9 and 11 from the previous reports. Part of this variation in the previous and current studies is due to methodological differences; however, it seems that ethnic differences should not be underestimated. There are very few studies from the geographic region of Iran; however, the reported cases from the countries such as Turkey are very similar to our findings.

## Introduction

Gastrointestinal stromal tumors (GISTs) are the most common mesenchymal tumors of the gastrointestinal (GI) tract (1% of all GI malignancies). Stomach and the proximal part of the small intestine are the most common locations of these tumors, but any part of the GI tract can be affected, including peritoneum, mesentery and occasionally omentum.^1^ They originate from the interstitial cells of Cajal (ICC) in the GI tract, in contrast to for example lieomyoma, which originate from smooth muscle cells.^2^

About 80-90% of GISTs show immunohistochemical staining for CD117 (c-KIT) and most of them contain activating mutations in the KIT receptor tyrosine kinase.^2^ About 10% of the GISTs contain mutations in PDGFRA (platelet derived growth factor receptor-alpha) and 7-10% of the GISTs are wild type for KIT and PDGFRA. The KIT mutations are mainly located in exon 9 and 11.^3^

The presence and absence of mutations of KIT in GISTs have predictive value for response to treatment and long-term prognosis.^4^ The frequency and type of KIT mutation has been reported from 80-90% in other countries, but there has been no published study from Iran. Therefore, in this research we have studied 50 cases of GISTs from different parts of the GI tract by histology, immunohistochemistry for c-KIT and molecular analysis of KIT mutations, to find out the relative frequency of this mutation in GISTs in Iran.

## Patients and Methods

During 5 years (2007-2012), we retrieved 50 cases of GISTs from the archives of the affiliated hospitals of Shiraz University of Medical Sciences. We excluded recurrent or treated cases; it means that all cases in this study have been primary diagnosis of GISTs. 

Demographic findings were extracted from the clinical charts. All pathology slides were reviewed and the diagnosis was confirmed, also the best slide and paraffin block was selected for both immunohistochemistry and DNA extraction for molecular analysis. 

Immunohistochemistry (IHC) for c-KIT was performed such that the slides were reviewed and the results were recorded. Immunohistochemistry for DOG-1 was performed, the slides were reviewed, and the diagnosis was confirmed as positive or negative. Table 1 shows the details of antibodies used for c-KIT and DOG-1.

**Table 1:** Characteristics of antibodies used for c-KIT and DOG-1

**Company**	**Detection method**	**Antigen retrieval**	**Concentration**	**Antibody**
NOVACASTRA	Envision	Citrate buffer pH=6	1/200	DOG1
DAKO	Envision	Citrate buffer pH=6	1/1000	C-KIT

Tumor DNA was extracted from the paraffin-embedded formalin fixed GISTs according to the manufacturer’s instruction (QIAmp DNA FFPE tissue kit). Molecular analysis of KIT mutation was performed by semi-nested PCR and Sanger DNA sequencing according to the previous reports with little modification. The material used in the PCR was very similar to usual PCR; note the details of the primer sequences as published by Hostein et al.^5^^,^^6^

## Results

During the study period, there were 50 cases of GIST, 29 patients were male and 21 patients were females, aged 33-82 years old (58.7±14). Thirty-three tumors originated from the stomach, 4 from the ileum, 2 from the jejunum, 2 from the duodenum, 1 from the ascending colon, 1 from the rectum, 1 from the retro-peritoneal area and 6 of the tumors were from the intra-abdominal cavity. 

**Figure 1:**
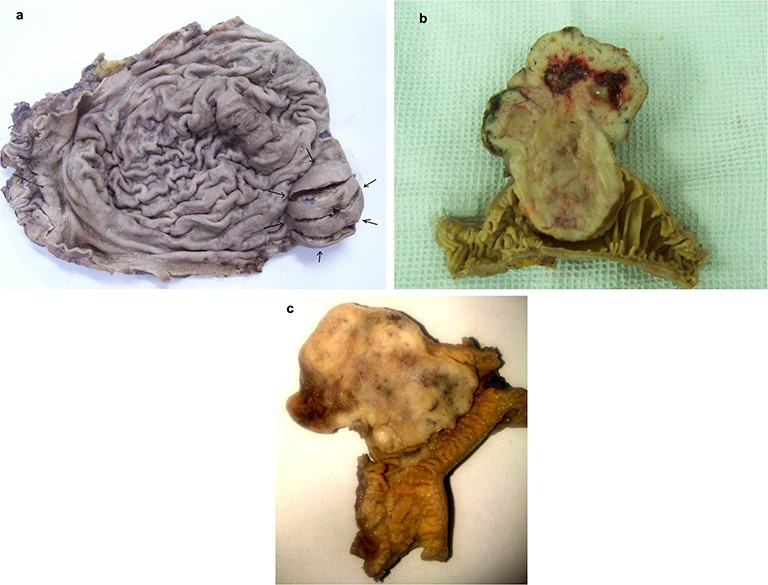
a) Gross of gastric GIST (arrows) b) Gross of small intestinal GIST c) Gross of large intestinal GIST.

Macroscopic evaluation of the tumors showed 22 tumors larger than 10 cm while 13 cases were between 5 and 10 cm, 10 tumors were smaller than 5 cm and only 4 cases were smaller than 2 cm, figure 1.

**Figure 2:**
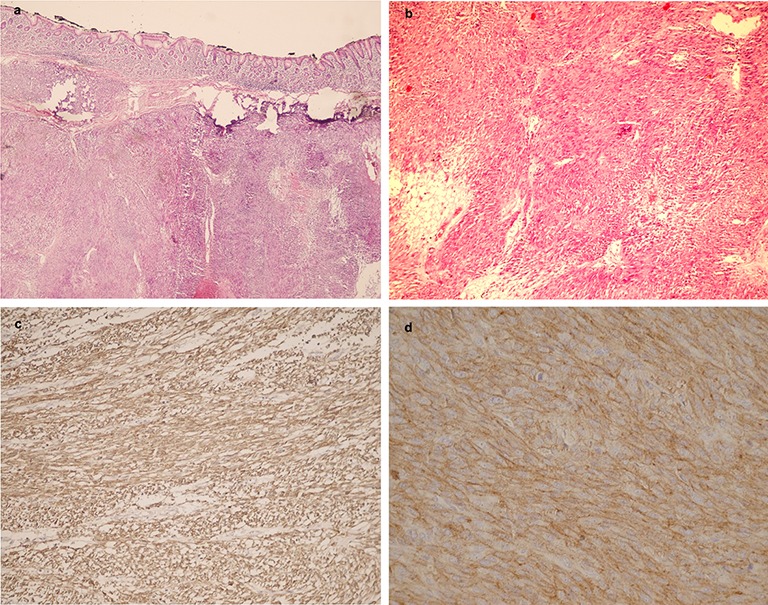
a) Sections from the stomach showing spindle cell tumor (H&E ×300) b) Sections from GIST show spindle cell tumor with low mitosis and no atypia (H&E ×250) c) Immunohistochemical staining of a GIST for c-Kit d) Immunohistochemical staining of a GIST for DOG-1.

In microscopic evaluation, 29 cases had spindle morphology, 7 tumors were epithelioid and the remainder were mixed epithelioid and spindle cell. Mitotic rate was less than 5/50 HPF in 31 cases, and over 5/50 HPF in 19 cases, figure 2.

IHC for c-KIT was positive in all of the 50 cases, DOG-1 was positive in 39 (78%) cases and 11 (22%) of the cases were negative for DOG-1.

Molecular analysis of KIT mutation showed 17 cases with wild type KIT and 33 cases (66%) with mutation either in exon 9 or in exon 11. The mutation of exon 9 was detected in 11 (22%) cases, while 29 (58%) cases had mutation of exon 11. In seven cases, both exon 11 and exon 9 mutations were detected at the same time (14%). Table 2 shows the detail of different types of mutations detected in these 50 cases. The most common type of mutation was deletion, which is shown in table 2.

**Table 2:** Characteristics of different types of mutation according to age, location and mutations

**Location**	**Numbers**	**Age**	**Size**	**Mutation in exon 9**	**Mutation in exon 11**
Stomach	33	40-82	1.8-20	5	24
Small intestine	8	40-77	3-17	1	2
Large intestine	2	43-49	1-15	0	0
Intra-abdominal	6	43-68	8-25	4	3
Retroperitoneal	1	52	10	1	0

## Discussion

Gastrointestinal stromal tumors (GISTs) originate from the interstitial cells of Cajal (ICC).^2^ c-KIT (CD117) is a type III receptor tyrosine kinase that is involved in the development and maintenance of ICCs. Loss of function KIT mutations causes gastrointestinal abnormalities due to loss of ICC. ICCs are unique pace maker cells that are located between autonomic nervous system and the muscular wall of the bowel and are responsible for coordinating peristalsis.^7^^,^^8^

In GIST tumors, activation of KIT leads to the activation of the KIT receptor and signal transduction cascades, resulting in signaling for cell proliferation and survival.^9^

The percentage of GISTs that have been reported to be KIT mutation positive varies from 57%^10^ to 92%.^11^ From the total of 50 studied cases, 33 (66%) had mutations while the remaining 17 cases (34%) were free of mutation in two common exon 9 and exon 11. The exon 9 mutation was detected in 11 cases (22%) while 26 cases (58%) were reported to have exon 11 mutations. It was in seven cases (14%) where both exon 11 and exon 9 mutations were detected at the same time. Generally, the results indicate that the frequency of KIT mutation positivity is consistent with some of the previous reports, especially in the Asian countries such as Japan.^12^

On the other hand, the overall mutation rate in our study was less than the frequencies observed in a population study from Norway (75.3%), from the reports of the European Organization for Research and Treatment of Cancer (EORTC) (83.6%) and also the United States (83%, and 92%).^11^^,^^13^^,^^14^ This frequency rate was also lower than Iceland (87.5%)^15^ and Panama (77%).^16^

In our study, KIT exon 11 mutants accounted for 58% of GISTs, which is in relative agreement with the study from Norway (65.2%) and Germany (60.9%).^13^^,^^17^ However, regarding the exon 9 mutations, the frequency of exon 9 mutations was 22 percent in our study, which was much lower than the above mentioned studies.^13^^,^^17^ Such disagreement in the frequency of exon 9 mutations could be due to racial differences. In the study of Heinrich et al.,^14^ those values were 72.9% and 8.2%, respectively. This study was conducted within phase III clinical trials; therefore, the discrepancy may reflect a difference in mutation profile observed due to referral material, with more overtly malignant GISTs enrolled to clinical trials.^18^Table 3 shows the comparison between our studies with some of the similar studies conducted on GISTs.

The variability regarding the incidence rate of KIT mutations might also be related to methodological differences among these retrospective studies. The type of tumor tissue available for DNA extraction (archival material versus frozen tissue), different techniques used for detecting KIT mutations such as using simple PCR product-length analysis, and a certain number of mutations such as point mutations would be missed.^19^ Whereas with the method of using denaturing high-pressure liquid chromatography, there would be a higher number of KIT mutations detected.^22^ Moreover, it has been proposed that the evaluation of only a restricted segment of the mutational hot spot in Juxta-Membranous region, detects a significantly lower rate of KIT mutations compared with a systematic sequencing of the entire c-DNA KIT coding sequence.^20^

Our report is the first one from Iran; and there are very few studies from our geographic region except for Turkey, which has a very similar finding to our results,^21^table 3.

**Table 3:** Comparison of our results with some of the previous reports according to the method and type of specimen

**Study**	**Method**	**Sample size**	**Total mutation**	**Mutation of exon 11**	**Mutation of exon 9**
USA, 2001^14^	-PCR (FFPE tissue) -DNA sequencing	48	44 (92%)	39 (71%)	6 (13%)
Iceland, 2010^15^	-PCR (FFPE tissue) -DNA sequencing	56	49 (87.5%)	43 (76.8%)	6 (10.7%)
Panama, 2011^16^	-PCR (FFPE tissue) -DNA sequencing	39	30 (77%)	27 (69%)	9 (8%)
Germany, 2011^17^	-PCR (FFPE tissue) -DNA sequencing	87	61 (70.1%)	53 (60.9%)	7 (8.1%)
Poland, 2011^18^	-PCR (FFPE tissue) -HPLC & sequencing	427	351 (82.2%)	261 (61.1%)	31 (7.3%)
Japan, 1987-1997^19^	-PCR (FFPE tissue) -DNA sequencing	124	71 (57%)	71 (57%)	_
China, 2012^20^	-PCR (fresh tissue) -DNA sequencing	49	_	29 (60%)	6 (13%)
Turkey, 2013^21^	-PCR (FFPE tissue) -DNA sequencing	60	41 (68.4%)	28 (46.7%)	5 (8.3%)
Current Study	-PCR (FFPE tissue) -DNA sequencing	50	33 (66%)	26 (58%)	11 (22%)

As we have shown in our previous report about immunohistochemistry of c-KIT in GIST,^23^ regarding molecular aspects in this tumor, comparing the results of our study with previous reports shows that a variety of factors could be mentioned as the possible source of variable KIT mutations incidence reports, such as racial differences, study population, and methodological variations.

## Conclusion

KIT mutation analysis showed frequency relatively similar to other parts of the world. 

## References

[1] Miselli F, Millefanti C, Conca E, Negri T, Piacenza C, Pierotti MA (2008). PDGFRA immunostaining can help in the diagnosis of gastrointestinal stromal tumors. Am J Surg Pathol.

[2] Jung SH, Suh KS, Kang DY, Kang DW, Kim YB, Kim ES (2011). Expression of DOG1, PDGFRA, and p16 in Gastrointestinal Stromal Tumors. Gut Liver.

[3] Origone P, Gargiulo S, Mastracci L, Ballestrero A, Battistuzzi L, Casella C (2013). Molecular characterization of an Italian series of sporadic GISTs. Gastric Cancer.

[4] Garces-Albir M, Marti-Obiol R, Lopez-Mozos F, Calabuig-Farinas S, Navarro-Ros S, Ortega-Serrano J (2012). Results on prognostic value of mutations in localized gastrointestinal stromal tumors (GIST) in one single center. Rev Esp Enferm Dig.

[5] Agaimy A, Terracciano LM, Dirnhofer S, Tornillo L, Foerster A, Hartmann A (2009). V600E BRAF mutations are alternative early molecular events in a subset of KIT/PDGFRA wild-type gastrointestinal stromal tumours. J Clin Pathol.

[6] Hostein I, Debiec-Rychter M, Olschwang S, Bringuier PP, Toffolati L, Gonzalez D (2011). A quality control program for mutation detection in KIT and PDGFRA in gastrointestinal stromal tumours. J Gastroenterol.

[7] Rubin BP (2006). Gastrointestinal stromal tumours: an update. Histopathology.

[8] Miettinen M, Lasota J (2006). Gastrointestinal stromal tumors: review on morphology, molecular pathology, prognosis, and differential diagnosis. Arch Pathol Lab Med.

[9] Nilsson B, Bumming P, Meis-Kindblom JM, Oden A, Dortok A, Gustavsson B (2005). Gastrointestinal stromal tumors: the incidence, prevalence, clinical course, and prognostication in the preimatinib mesylate era--a population-based study in western Sweden. Cancer.

[10] Martin J, Poveda A, Llombart-Bosch A, Ramos R, Lopez-Guerrero JA, Garcia del (2005). Deletions affecting codons 557-558 of the c-KIT gene indicate a poor prognosis in patients with completely resected gastrointestinal stromal tumors: a study by the Spanish Group for Sarcoma Research (GEIS). J Clin Oncol.

[11] Rubin BP, Singer S, Tsao C, Duensing A, Lux ML, Ruiz R (2001). KIT activation is a ubiquitous feature of gastrointestinal stromal tumors. Cancer Res.

[12] Taniguchi M, Nishida T, Hirota S, Isozaki K, Ito T, Nomura T (1999). Effect of c-kit mutation on prognosis of gastrointestinal stromal tumors. Cancer Res.

[13] Steigen SE, Eide TJ, Wasag B, Lasota J, Miettinen M (2007). Mutations in gastrointestinal stromal tumors--a population-based study from Northern Norway. APMIS.

[14] Heinrich MC, Owzar K, Corless CL, Hollis D, Borden EC, Fletcher CD (2008). Correlation of kinase genotype and clinical outcome in the North American Intergroup Phase III Trial of imatinib mesylate for treatment of advanced gastrointestinal stromal tumor: CALGB 150105 Study by Cancer and Leukemia Group B and Southwest Oncology Group. J Clin Oncol.

[15] Tryggvason G, Hilmarsdottir B, Gunnarsson GH, Jonsson JJ, Jonasson JG, Magnusson MK (2010). Tyrosine kinase mutations in gastrointestinal stromal tumors in a nation-wide study in Iceland. APMIS.

[16] Mendoza Y, Singh C, Castillo Mewa, Fonseca E, Smith R, Pascale JM (2011). Beginning of personalized medicine in Panama: Molecular and pathological characteristics of gastrointestinal stromal tumors from archival paraffin-embedded tissue. Oncol Lett.

[17] Daniels M, Lurkin I, Pauli R, Erbstosser E, Hildebrandt U, Hellwig K (2011). Spectrum of KIT/PDGFRA/BRAF mutations and Phosphatidylinositol-3-Kinase pathway gene alterations in gastrointestinal stromal tumors (GIST). Cancer letters.

[18] Wozniak A, Rutkowski P, Piskorz A, Ciwoniuk M, Osuch C, Bylina E (2012). Prognostic value of KIT/PDGFRA mutations in gastrointestinal stromal tumours (GIST): Polish Clinical GIST Registry experience. Ann Oncol.

[19] Merkelbach-Bruse S, Dietmaier W, Fuzesi L, Gaumann A, Haller F, Kitz J (2010). Pitfalls in mutational testing and reporting of common KIT and PDGFRA mutations in gastrointestinal stromal tumors. BMC Med Genet.

[20] Qiu C, Liu X, Bai C, Ma DL (2012). The expression of KIT receptor dimers in gastrointestinal stromal tumors independent of c-kit mutation and SCF expression is associated with high-risk stratification. Oncol Lett.

[21] Calibasi G, Baskin Y, Alyuruk H, Cavas L, Oztop I, Sagol O (2014). Molecular analysis of the KIT gene in gastrointestinal stromal tumors with novel mutations. Appl Immunohistochem Mol Morphol.

[22] Corless CL, McGreevey L, Haley A, Town A, Heinrich MC (2002). KIT mutations are common in incidental gastrointestinal stromal tumors one centimeter or less in size. Am J Pathol.

[23] Geramizadeh B, Nosrati A (2006). Histological and immunological evaluation of gastrointestinal stromal tumors. Iran J Med Sci.

